# Blastoid mantle cell lymphoma presenting as acute leukemia: a case report

**DOI:** 10.11604/pamj.2021.39.150.28903

**Published:** 2021-06-28

**Authors:** Amol Dongre, Niraj Kanchankar, Mahesh Deshmukh, Dilip Wasnik, Trupti Dongre

**Affiliations:** 1 Department of Medical Oncology, Alexis Multispeciality Hospital, Nagpur, Maharashtra, India,; 2 Department of Radiology, Alexis Multispeciality Hospital, Nagpur, Maharashtra, India,; 3 Department of Pathology, Alexis Multispeciality Hospital, Nagpur, Maharashtra, India,; 4 Department of Anaesthesia, Alexis Multispeciality Hospital, Nagpur, Maharashtra, India,; 5 Department of Pathology, Narendra Kumar Prasadrao (NKP) Salve Institute of Medical Sciences and Research Centre and Lata Mangeshkar Hospital, Nagpur, Maharashtra, India

**Keywords:** Blastoid variant, mantle cell lymphoma, leukemia, case report

## Abstract

Blastoid variant of mantle cell lymphoma (MCL) is an aggressive and incurable form of B-cell lymphoma. Various chemo-immunotherapy regimens have been used with a limited success. Autologous stem cell transplant (ASCT) is being increasingly recommended in first remission for patients with blastoid variant MCL. Here, we describe the case of a 45-year-old lady suffering from blastoid variant of MCL. Patient was administered R-CHOP and underwent autologous stem cell transplant (ASCT). Currently, patient is disease-free and is on a regular follow up for 26 months now. This is an extremely rare case documented in the Indian literature with a long survival post-transplant.

## Introduction

Mantle cell lymphoma (MCL) is a rare, aggressive and incurable form of B-cell lymphoma due (11;14)(q13;q32) genetic translocation in the naïve B-cells resulting in over-expression of cyclin D1 [1,2]. Blastoid mantle cell lymphoma refers to a subgroup of classical MCL which are even more aggressive due to the acquisition of additional molecular/cytogenetic mutations [1,3]. Blastoid variant constitutes about 10-20% of all cases of MCL [3]. Even after decades of research and improved biological understanding, these group of patients continue to have poor prognosis [2]. In the absence of no standard treatment protocol for MCL, patients are treated with various chemotherapy (CT) regimens, immunotherapy and autologous stem cell transplantation (ASCT) [1-3]. Here, we describe a case of a middle aged female with blastoid variant of MCL, treated successfully with chemotherapy (R-CHOP regimen) followed by autologous stem cell transplantation (ASCT) with a very good prognosis.

## Patient and observation

**Patient information:** a 45-year-old Indian female, with no known co-morbidities, presented with fever, generalised weakness, easy fatigability and swelling in the neck for the past 2-3 weeks. There was no history of hospitalization prior to this presentation. No similar history was present in any of her family members or of any other chronic illnesses.

**Clinical findings:** she was vitally stable. On physical examination, bilateral cervical lymph node enlargement was present along with massive splenomegaly (24 cm below the left costal margin) and hepatomegaly (10cm below the right costal margin). Examination of other systems was unremarkable.

**Figure 1:**
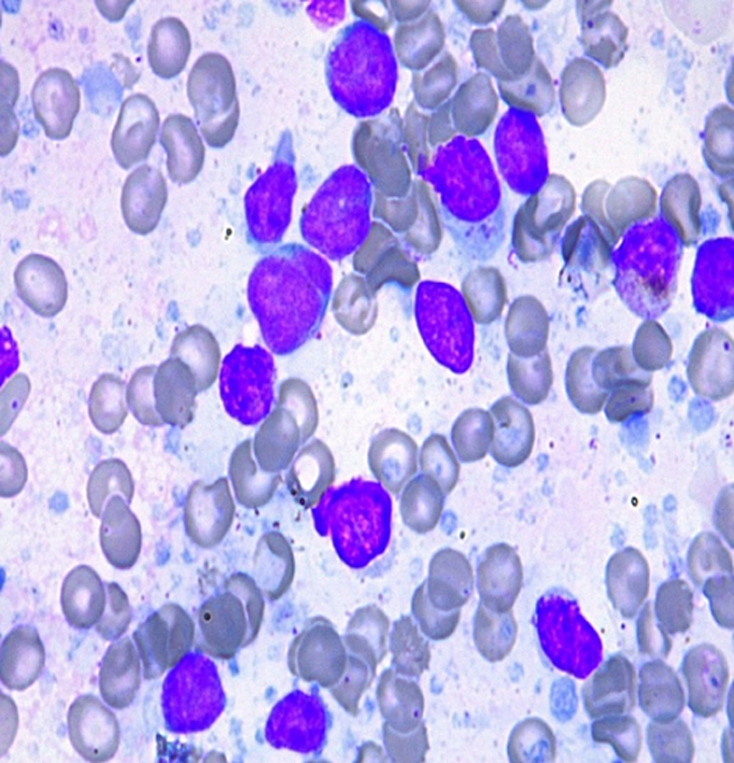
atypical lymphocytosis seen on bone marrow aspirate

**Figure 2:**
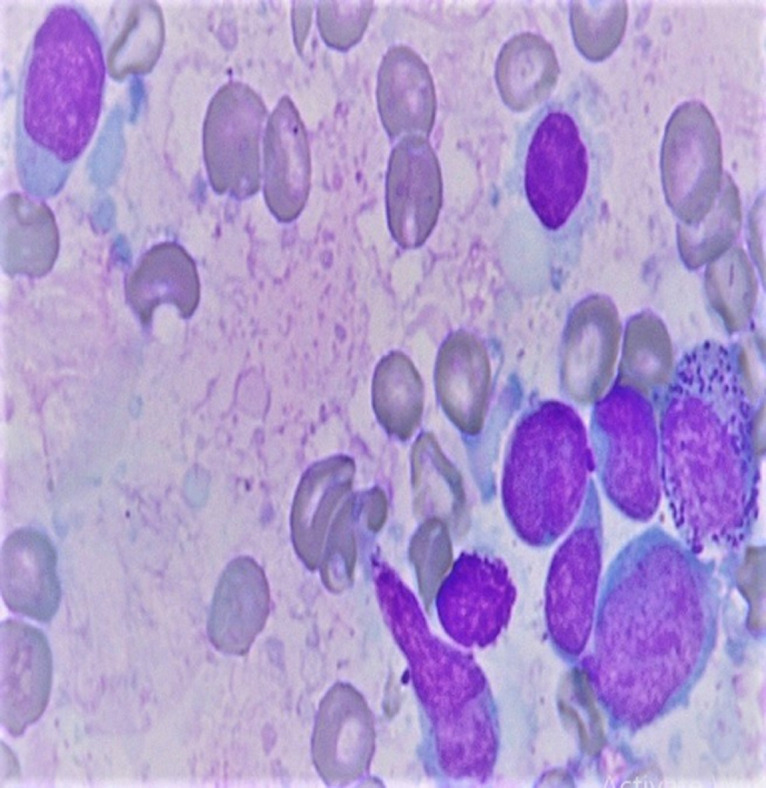
magnified image of atypical lymphocyte showing high N:C ratio with scant cytoplasm and dispersed nuclear chromatin

**Diagnostic assessment:** haemogram showed pancytopenia (haemoglobin-9.1gm%, total leukocyte count-1280 cells/μL, platelet count-63,000/μL) with peripheral smear showing 23% abnormal cells resembling lymphoblasts. Serum lactate dehydrogenase (LDH) levels were within limits. Liver and kidney functions were within normal limits. Computer tomography showed a generalized lymphadenopathy (cervical, axillary, retroperitoneal and inguinal) with the largest node measuring 6 x 5cm. Bone marrow aspiration performed showed 37% atypical lymphoid cells with a high N: C ratio with scant cytoplasm and dispersed nuclear chromatin with few cells showing irregular nuclear margins and inconspicuous nucleoli (Figure 1, Figure 2). Furthermore, trephine biopsy showed marked interstitial and focal nodular infiltration by those atypical lymphoid cells. Although, the lymphoblasts resembled as those seen in leukemia/ lymphoma, analysis was performed to further characterize these blast cells. Flow cytometry showed brightly CD45 positive cells but CD34 negative, thus, confirming that the atypical cells were mature lymphoid cells rather than blasts. Further, immunophenotyping of these atypical mature lymphoid cells showed positive B cell markers (CD5, CD19, CD20, CD22, CD79a) with surface kappa light chain restriction. In addition, the atypical cells expressed CD10. The aspirate was negative for other T-cell, myeloid and monocytic markers. Cyclin D1 was positive on immunohistochemistry in >10% of the atypical cells obtained from bone marrow biopsy. A diagnosis of mantle cell lymphoma (MCL) was made. Differential diagnosis considered at this stage were: small lymphocytic lymphoma, marginal zone lymphoma and follicular lymphoma. Immunophenotyping and flow cytometry had conclusively established this case to be blastoid variant of mantle cell lymphoma.

**Therapeutic intervention:** she was managed with chemo-immunotherapy. She received 6 cycles at 3 week intervals: Rituximab (375 mg/m^2^), cyclophosphamide (750 mg/m^2^), doxorubicin (50 mg/m^2^), vincristine (1.5 mg/m^2^) and prednisone (100mg; once daily for 5 days) (R-CHOP) regimen. She achieved complete remission after 4.5 months of the above R-CHOP regimen. As MCL is known for its aggressive nature and high risk of relapse, we decided to perform ASCT. She underwent ASCT in first complete remission with BEAM (carmustin, etoposide, cytarabine and melphalan) as a conditioning regimen. Post-transplant period was uneventful. Gradually, hemogram improved with normalisation of total leukocyte counts and platelet count. No atypical lymphocytes were seen. A repeat bone marrow examination and positron emission tomography–computed tomography (PET-CT) showed no abnormal findings. Thus, a complete metabolic and morphologic remission was achieved.

**Follow-up and outcome:** currently, on follow-up, after 26 months of ASCT, she is healthy with no active complaints or abnormal investigations. Further plan includes surveillance blood counts, LDH and 6 monthly imaging for disease recurrence.

## Discussion

The present case constitutes a rather infrequently reported clinical presentation of blastoid variant of mantle cell lymphoma (MCL) being managed very effectively with R-CHOP regimen followed by autologous stem cell transplantation (ASCT). Mantle cell lymphoma is classified into 2 clinically indolent variants: classical MCL and leukemic non-nodal MCL. The classic MCL has further 2 variants: pleomorphic and blastic variant. High proliferation index and unfavourable prognosis is common to both these variants [1,3]. Blastoid MCL affects male gender predominantly. It is commonly detected in an advanced stage among individuals in their sixth decade of life [3]. Lymph nodes are primarily affected with other organs being involved such as bone marrow, spleen [4]. Clinically, it presents with hepatosplenomegaly, generalised lymphadenopathy, bone marrow involvement with systemic symptoms such as weight loss, fever, and night sweats being present in upto 50% of patients [4]. Waldeyer´s ring and gastrointestinal tract are frequently involved extra-nodal sites [4]. Our patient had a similar clinical presentation with no extra-nodal involvement. Leukemic presentation of blastoid MCL is an exceedingly uncommon event. In addition, differentiating blastoid MCL from lymphoblastic lymphoma and centroblastic large cell lymphoma is extremely difficult solely on the basis of morphology, necessitating the use of additional analytical techniques such as immunophenotyping (IP) and molecular studies.

Certain histological and immunophenotypical features are frequently associated with the blastoid variant. Blastoid MCL pattern of growth is diffuse, less often nodular, rarely exhibiting the mantle zone pattern. In addition, an “In situ” pattern of blastoid MCL is not seen [1,3,4]. The cells resemble lymphoblasts with more dispersed chromatin and have a very high mitotic activity (≥20-30/10 HPF) as was observed in our case. Variability of immunophenotyping is frequently observed. Cyclin D1 and CD20 positivity are almost always seen [5]. CD5 inexpression is seen to occur more commonly than the classical MCL [6]. Blastoid variant also has high proliferation index (Ki-67 index) [5]. Immunophenotypic variants are not uncommon in MCL wherein usually blastoid variants can be CD10 positive (8%), which was true in the present case as well. The differential diagnosis includes acute lymphoblastic leukemia (ALL), leukemic phase of large cell lymphoma and B cell prolymphocytic leukemia (PLL). Immunophenotypic (IP) studies are extremely helpful in differentiating among the various differentials. IP study in ALL shows CD34, TdT expression while surface Ig and cyclin D1 are negative. Cyclin D1 is not expressed in large cell lymphoma as well. B-PLL may only be differentiated by the presence of (11;14) translocation and lack of cyclin D1 expression.

Various clinical and biologic features are used for prognostication in MCL. Mantle cell lymphoma international prognostic index (MIPI) is the most frequently used prognostic index for patients with MCL. It is based on 4 independent prognostic factors (age, performance status, LDH levels, and total leukocyte counts) [7,8]. Ki-67 index was found to be superior to using cytological and growth pattern for prognosis in MCL, hence, a modified combination of MIPI and Ki-67 index reflects a strong prognostic discriminative capacity [9]. R-CHOP is the most commonly used chemotherapy regimen in blastoid MCL. The addition of rituximab has increased the response rates and time for treatment failure [3]. Autologous stem cell transplantation prognosis is differential with some group of patients greatly benefiting while others may not benefit at all [10]. Group of population with MCL with potential benefit from ASCT inludes: blastoid/pleomorphic morphology, high MIPI score, less intense induction regimens (R-CHOP, BR) and no cytarabine in induction [10]. Our patient underwent ASCT after 6 cycles of R-CHOP regimen. Our case emphasizes the heterogeneity observed in blastoid MCL cases wherein in spite of being an advanced stage disease, she responded quite well. Although previously regarded as a disease with generally poor outcome, this case outlines the heterogeneous behaviour of B-MCL, and for this reason, attempts should be made to document and identify features that may influence prognosis.

## Conclusion

In conclusion, our case highlights that although the blastoid variant of MCL is a rare type of NHL with an overall poor prognosis, ASCT is a potentially curative therapy in patients with blastoid MCL.
